# The Rapid Cytological Process of Grain Determines Early Maturity in Weedy Rice

**DOI:** 10.3389/fpls.2021.711321

**Published:** 2021-08-31

**Authors:** Can Zhao, Wenrong Xu, Hewei Li, Weimin Dai, Zheng Zhang, Sheng Qiang, Xiaoling Song

**Affiliations:** Weed Research Laboratory, College of Life Sciences, Nanjing Agricultural University, Nanjing, China

**Keywords:** cultivated rice, endosperm cells, cytological process, antioxidative enzyme system, weedy rice

## Abstract

Shorter grain-filling period and rapid endosperm development endow weedy rice (WR) with early maturity compared to cultivated rice (CR). However, the role of the cytological features and antioxidative enzyme system during grain development are largely unexplored. We selected four biotypes of WR and their associated cultivated rice (ACR) types from different latitudes to conduct a common garden experiment. The difference in the cytological features of endosperm between WR and ACR was compared by chemical staining, and the cell viability and nuclear morphometry of endosperm cells were observed by optical microscopy. Furthermore, antioxidative enzyme activity was measured during grain filling. Anatomic observation of endosperm shows that the development process of endosperm cell in WR was more rapid and earlier than that in ACR. The percentage of degraded nuclei of WR was 2–83% more than that of ACR. Endosperm cells in WR lost viability 2–6 days earlier than those in ACR. The antioxidant enzyme activity of WR was lower than that of ACR during grain filling. The ability of WR to scavenge reactive oxygen species (ROS) was weaker than that of ACR, which may contribute to the rapid cytological process in the endosperm cells of WR. The rapid cytological process and weaker ability to scavenge ROS in endosperm cells may contribute to early maturity in WR.

## Introduction

Weedy rice (*Oryza sativa* L. f. *spontanea*, WR) has become one of the most harmful weeds in paddy fields worldwide (Azmi and Karim, 2008; Burgos et al., 2014; Kraehmer et al., 2016; Zhao et al., 2018, 2020). It belongs to the same genus as cultivated rice (*O. sativa* L., CR) and has AA nuclear chromosomes. Recently, it has been reported that WR originated from CR and experienced a strong genetic bottleneck effect during its evolution in China (Li et al., 2017; Qiu et al., 2017). Both WR and CR share similar morphological and physiological traits, which make controlling WR very difficult compared to other weeds (Chauhan, 2013). WR has many morphological and physiological characteristics related to weediness, such as a rapid growth rate, high phenotypic plasticity, early maturity, seed shattering, long seed dormancy, and longevity, which facilitate seed dispersal and persistence in paddy fields (Azmi and Karim, 2008; Burgos et al., 2014; Dai et al., 2014, 2017; Zhao et al., 2018, 2020). These characteristics contribute to its competitive advantage over CR in rice production areas globally (Delouche et al., 2007).

Among these weediness characteristics, the early maturity of WR contributes to its escape from harvesting (Sánchez-Olguín et al., 2007; Zhao et al., 2018). Our previous study found that the shorter grain-filling period promotes early maturity in WR compared with the associated cultivated rice (ACR) (Zhao et al., 2018). Furthermore, the rapid development of endosperm cells and starch grains leads to the shorter grain-filling period of WR (Zhao et al., 2020). The endosperm accounts for 91–92% of the total weight of rice grains. It stores a large amount of starch, which serves as the primary carbohydrate component in human and livestock diets, and a small amount of protein (Sabelli and Larkins, 2009). The endosperm cell development of rice is divided into four stages: the coenocyte stage, cellularization stage, differentiation stage, and maturation stage during grain filling (Olsen, 2004). During the development of endosperm cells, the nucleus is the first to die, e.g., nuclear deformation, nuclear membrane rupture, chromatin condensation, and nucleocytoplasmic leakage (Young and Gallie, 1999; Wei et al., 2002). However, the difference of cytological process in endosperm cells between WR and ACR is unclear.

During the development of endosperm cells, the accumulation of storage compounds is accompanied by degradation and death of endosperm cells (Sabelli and Larkins, 2009; Wang et al., 2012; Kobayashi et al., 2013; Li et al., 2018). Antioxidative enzyme activity is closely related to the development of rice endosperm cells because reactive oxygen species (ROS), such as hydrogen peroxide (H_2_O_2_), superoxide anions (O^2–^), hydroxyl radicals (.OH), and singlet oxygen (^1^O_2_), play an important role in the occurrence of plant cell development (Halliwell, 2006). ROS upset cell metabolism through oxidative damage to lipids, proteins, and nucleic acids, resulting in plant growth and natural senescence. Plants protect cells from ROS injury by means of an antioxidant enzyme system [e.g., SOD, catalase (CAT), peroxidase (POD)] and antioxidant materials [e.g., ascorbic acid (ASA) and glutathione (GSH)] that scavenge ROS (Nunez et al., 2003; Nguyen and Donaldson, 2005; Corpas et al., 2006; Zhang et al., 2015). The activity of antioxidant enzymes, such as SOD and CAT, decreases, and intracellular levels of ROS rapidly rise during the plant cell development and natural senescence (Fath et al., 2001; Pulido et al., 2009). Differences in antioxidant enzyme activity between WR and ACR endosperm cells have not been reported.

The cytological process of endosperm development in rice affects endosperm growth and cell death, then determines the growth period of rice (Kobayashi et al., 2013). There are many reports about the cytological process of CR, but research on the cytological process contributing to rapid grain filling in WR has not been reported. In the present study, we used Steedman’s wax embedded sections, 4′,6-diamidino-2-phenylindole (DAPI) staining, Evans blue staining, and 2,3,5-tryphenyl tetrazolium chloride (TTC) staining to compare and analyze the differences in the cytological process in endosperm cells and measured the antioxidative enzyme activity between WR and ACR during grain filling. Our objectives were to reveal the differences in the cytological process and clarify the difference in antioxidant enzyme activity during endosperm cells development. Our results on the differences in the cytological mechanism of rapid grain development between WR and ACR may provide a theoretical basis for understanding the weedy characteristics of WR.

## Materials and Methods

### Experimental Location and Cultivation Methods

Field trials were established at Jiangpu Experimental Farm (118°37′E, 32°02′N), Nanjing Agricultural University, China, in the summer cropping seasons (from May to November) of 2015. According to our previous studies on the morphological characteristics of various WR accessions (Dai et al., 2014; Zhao et al., 2018, 2020), we selected four WR accessions of different geographic origins along with their associated cultivars at the collection site. The characteristics of the four WR and associated cultivar biotypes are listed in Table 1. The materials were previously described by Zhao et al. (2018). The local cultivars included Nangeng-5055 (TZCR) and Zhong Lian Hui-950 (YZCR), which are both widely cultivated in Jiangsu Province; Dangeng-17 (DDCR), which is widely planted in Dandong City of Liaoning Province; and Yue Xin Zhan-2 (MMCR), which is from Maoming City of Guangdong Province. The experimental plots consisted of a 20 m^2^ plot for each of the WR and CR accessions, 50 cm spaces between the adjacent plots, sowing distances of 30 cm × 15 cm, and a design of 20 rows × 20 columns. The individuals of each accession were planted in separate plots with three replications. The phenotype of each WR and CR was showed in Supplementary Figure 1.

**TABLE 1:** Characteristics of the representative weedy rice (*Oryza sativa* L. f. *spontanea*) and cultivated rice (*Oryza sativa* L.) accessions used in the experiments.

Types	District	Population number of rice	Cultivar or accession	Origin (city, province)	Pericarp color	Subspecies	Longitude	Latitude
Cultivated rice	Northeast China	WRLN004R1	Dangeng-17 (DDCR)	Dandong, Liaoning	White	*Typical japonica*	124°17′E	39°58′N
	Eastern China	WRJS023R1	Zhong Lian Hui-950 (YZCR)	Yangzhou, Jiangsu	White	*Typical indica*	119°20′E	32°20′N
	Eastern China	WRJS013R1	Nangeng-5055 (TZCR)	Taizhou, Jiangsu	White	*Typical japonica*	119°57′E	32°26′N
	Southern China	WRGD008R1	Yue Xin Zhan-2 (MMCR)	Maoming, Guangdong	White	*Typical indica*	110°50′E	21°40′N
Weedy rice	Northeast China	WRLN004	DDWR	Dandong, Liaoning	Red	*Japonica*	124°17′E	39°58′N
	Eastern China	WRJS023	YZWR	Yangzhou, Jiangsu	Red	*Indica*	119°20′E	32°20′N
	Eastern China	WRJS013	TZWR	Taizhou, Jiangsu	Red	Indica clinous	119°57′E	32°26′N
	Southern China	WRGD008	MMWR	Maoming, Guangdong	Red	*Indica*	110°50′E	21°40′N

*TZWR, weedy rice from Taizhou; TZCR, cultivated rice from Taizhou; YZWR, weedy rice from Yangzhou; YZCR, cultivated rice from Yangzhou; MMWR, weedy rice from Maoming; MMCR, cultivated rice from Maoming; DDWR, weedy rice from Dandong; DDCR, cultivated rice from Dandong.*

### Sample and Data Collection

#### Sample Preparation for Evaluating Endosperm Nuclear Morphology of WR and ACR

Each panicle from 10 plants that headed on the same day were chosen and tagged in each plot. The flowering date of each upper spikelet on the tagged panicles was recorded and marked. The marked spikelets were sampled at 3, 5, 7, and 9 days post anthesis (DPA). Then 10 grains of each WR and CR were collected from these spikelets at 3, 5, 7, and 9 DPA. Both ends of the grains were removed, leaving the 2–3 mm middle portion. The samples of grains were fixed in 2.5% glutaraldehyde (Sigma Chemical Company, St. Louis, MO, United States) in 100 mM sodium phosphate buffer (pH 7.2) for 24 h at room temperature and then overnight at 4°C. The samples of grains were rinsed with the 100 mM sodium phosphate buffer three times and then dehydrated in a concentration series of ethanol solutions. Then these samples of grains were embedded at 37°C in Steedman’s wax previously prepared from PEG 400 distearate (Sigma Chemical Company, St. Louis, MO, United States) and 1-hexadecanol (Sigma Chemical Company, St. Louis, MO, United States) (9:1) as described by Wei et al. (2009). Waxed grains were cut into approximately 8-μm-thick sections on a rotary microtome (Leica RM2235, Germany), mounted on slides coated with glycerol albumin, and then dewaxed in absolute ethanol. A drop of distilled water was deposited on the slide, the slices were floated on the distilled water for expansion, and the slices were baked overnight at 30°C. Then, they were dewaxed overnight, and continue to be dewaxed with absolute ethanol 1–2 times, for 2–3 h each time, and then dried naturally (He et al., 2002). Dewaxed glass slides containing grain tissues were stained with DAPI (1 μg/mL) (Sigma Chemical Company, St. Louis, MO, United States) and examined with a fluorescence microscope (Zeiss Discovery V20, Germany). Stained nuclei showed blue fluorescence with UV excitation. Nuclear morphology of endosperms of WR and CR was observed. The normal, deformed, and degraded nuclei in more than 100 endosperm cells of each WR and CR were numbered at 3, 5, 7, and 9 DPA.

#### Sample Preparation for Evaluating Endosperm Cell Viability

The grain samples were collected with the same methods as that in 2.2.1 at 3, 5, 7, 9, 11, 13, 15, 18, and 21 DPA. TTC staining and Evans blue staining were used to observe the process and pattern of cell death in endosperm cells. TTC stains viable cells or tissues, while Evans blue dye stains dead cells, which are indicated to be dead by specific stains used in viability assays, such as TTC or Evans blue (van Doorn et al., 2011). The TTC staining method was modified from Oberle and Watson (1953). Thin sections of grains were made by hand using sharp double-sided blades at different DPAs (3, 5, 7, 9, 11, 13, 15, 18, and 21 DPA) and stained in 0.5% (w/v) TTC (Aladdin, E104208-10 g, United States) for 30 min at 25°C. Stained sections were photographed with a stereomicroscope (Zeiss Discovery V20, Germany). At least five grains were observed. The Evans blue staining method was modified from Young and Gallie (1999). Grains at 3, 5, 7, 9, 11, 13, 15, 18, and 21 DPA were cut with sharp double-sided blades by hand and stained in 0.1% (w/v) Evans blue (Aladdin, E104208-10 g, United States) for 2 min. Stained sections were washed with water for 1 h and photographed with a stereomicroscope (Zeiss Discovery V20, Germany). At least five grains were observed for each WR and CR.

#### Measurements of CAT, POD, and SOD Activities

A total of 320–340 panicles that headed on the same day were chosen and tagged in each plot. The flowering date of each upper spikelet on the tagged panicles was recorded and marked. The marked spikelets were sampled at 3, 5, 10, 15, 20, 25, and 30 DPA. Grains were collected from the upper region of each spikelet. Then, grains from the same plot were combined to form one sample. Approximately 200 sampled grains of WR or CR for each plot were frozen in liquid nitrogen for 2 min before storage at −80°C to measure the activity of antioxidative enzymes.

Catalase activity was determined by following the consumption of H_2_O_2_ (extinction coefficient 39.4 mM^–1^ cm^–1^) at 240 nm for 3 min (Aebi, 1984). POD activity was assayed by the method described by Cakmak and Marschner (1992). SOD activity was determined by measuring its ability to inhibit the photochemical reduction of nitroblue tetrazolium (NBT) according to the method of Giannopolitis and Ries (1977).

### Data Analysis

When comparing the morphology of starch endosperm cell nuclei of WR and ACR, the independent-sample *t*-test (*P* < 0.05) was used. For the activity of antioxidative enzymes, the means were compared using the Duncan’s multiple range test (DMRT) (*P* < 0.05). All statistical analyses were conducted using the SPSS software package (18.0; SPSS Inc., Chicago, IL, United States), and graphs were generated using Origin 8.0 (OriginLab, Hampton, MA, United States).

## Results

### Nuclear Morphology of Endosperms of Weedy and Cultivated Rice

**FIGURE 1:**
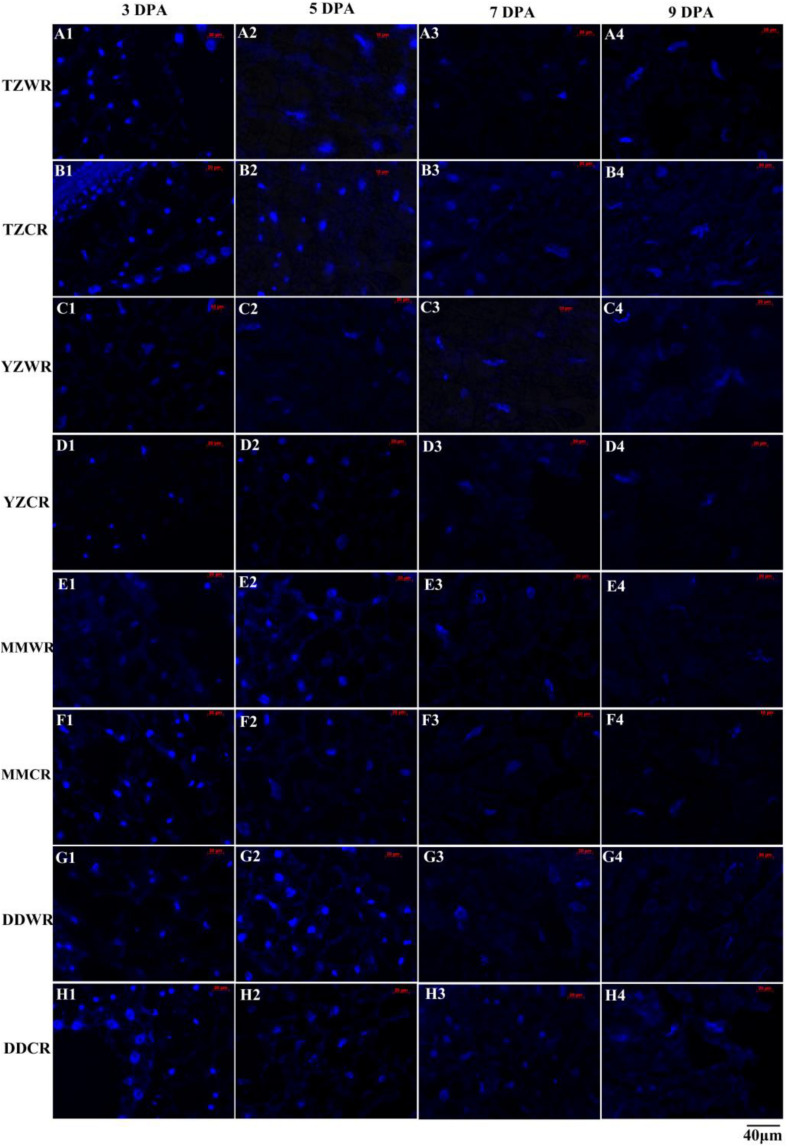
4′,6-Diamidino-2-phenylindole staining of endosperm nucleus in weedy and cultivated rice. **(A1–A4)**: weedy rice from Taizhou, TZWR; **(B1–B4)**: cultivated rice from Taizhou, TZCR; **(C1–C4)**: weedy rice from Yangzhou, YZWR; **(D1–D4)**: cultivated rice from Yangzhou, YZCR; **(E1–E4)**: weedy rice from Maoming, MMWR; **(F1–F4)**: cultivated rice from Maoming, MMCR; **(G1–G4)**: weedy rice from Dandong, DDWR; **(H1–H4)**: cultivated rice from Dandong, DDCR.

**FIGURE 2:**
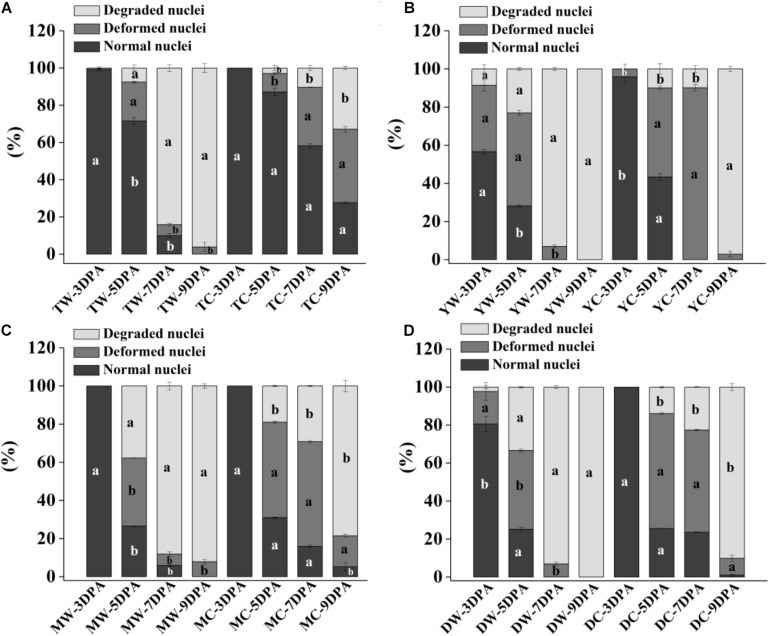
Proportion of normal, deformed, and degraded nuclei in endosperm cells of weedy and cultivated rice. **(A)** Weedy and cultivated rice from Taizhou; **(B)** weedy and cultivated rice from Yangzhou; **(C)** weedy and cultivated rice from Maoming; **(D)** weedy and cultivated rice from Dandong. TW, weedy rice from Taizhou; TC, cultivated rice from Taizhou; YW, weedy rice from Yangzhou; YC, cultivated rice from Yangzhou; MW, weedy rice from Maoming; MC, cultivated rice from Maoming; DW, weedy rice from Dandong; DC, cultivated rice from Dandong. DPA, days post-anthesis. Different lowercase letters indicate statistical significance for the comparison between weedy rice and its associated cultivated rice (independent-sample *t*-test, *P* < 0.05). The data are presented as mean ± SE (*n* = 3).

Endosperm cell development is often accompanied by degeneration of cell nuclei. DAPI is a highly sensitive and specific DNA fluorescent dye of the cell nucleus and chromosomes. The endosperm cell nuclei of WR and CR in the coenocyte or cellularization stages were small and regularly spherical at 3 DPA. Starch accumulated continuously in endosperm cells, and the nucleus of the starch endosperm was extruded, gradually deformed and disintegrated at 5–9 DPA (Figures 1, 2). Generally, the cytological process of endosperm cell of WR was faster than that of their CR (Figures 1, 2).

Morphological and statistical results of starch endosperm cell nuclei of WR and ACR (normal nuclei, deformed nuclei, and degraded nuclei) at 3, 5, 7, and 9 DPA are shown in Figures 1, 2. After DAPI staining, 100% of the nuclei of the endosperms of WR and CR in Taizhou were normal at 3 DPA. The percentage of normal nuclei of TZWR was 16% lower than that of TZCR at 5 DPA, and the percentages of deformed nuclei and degraded nuclei of TZWR were 11 and 5% higher than those of TZCR at 5 DPA, respectively. The percentages of normal nuclei and deformed nuclei of TZWR were 48 and 28% lower than those of TZCR at 7 and 9 DPA, respectively; however, the percentage of degraded nuclei of TZWR was 74 and 63% higher than that of TZCR at 7 and 9 DPA, respectively (Figures 1A1–A4,B1–B4,2A).

After DAPI staining, the percentage of normal nuclei of YZWR was 39 and 15% lower than that of YZCR at 3 and 5 DPA, respectively, and the percentages of deformed nuclei and degraded nuclei of YZWR were 2–31% higher than those of YZCR at 3 and 5 DPA. There were no normal nuclei in the endosperm cells of YZWR and YZCR, but the percentage of degraded nuclei of YZWR was 83 and 3% higher than that of YZCR at 7 and 9 DPA, respectively (Figures 1C1–C4,D1–D4,2B).

The endosperm cells of MMWR and MMCR showed normal nuclei at 3 DPA. From 5 to 9 DPA, the percentage of normal nuclei and deformed nuclei of MMWR was 4–49% lower than that of MMCR; however, the percentage of degraded nuclei of MMWR was 14–59% higher than that of MMCR (Figures 1E1–E4,F1–F4,2C).

The percentages of deformed nuclei and degraded nuclei were 18 and 2% at 3 DPA in DDWR, respectively. However, all nuclei were normal in DDCR at 3 DPA. From 5 to 9 DPA, the percentages of normal nuclei and deformed nuclei of DDWR were 1–47% lower than those of DDCR, and the percentage of degraded nuclei of DDWR was 10–70% higher than that of DDCR (Figures 1G1–G4,H1–H4,2D).

### Endosperm Cell Viability of Weedy and Cultivated Rice

#### Evans Blue Dye Assay

**FIGURE 3:**
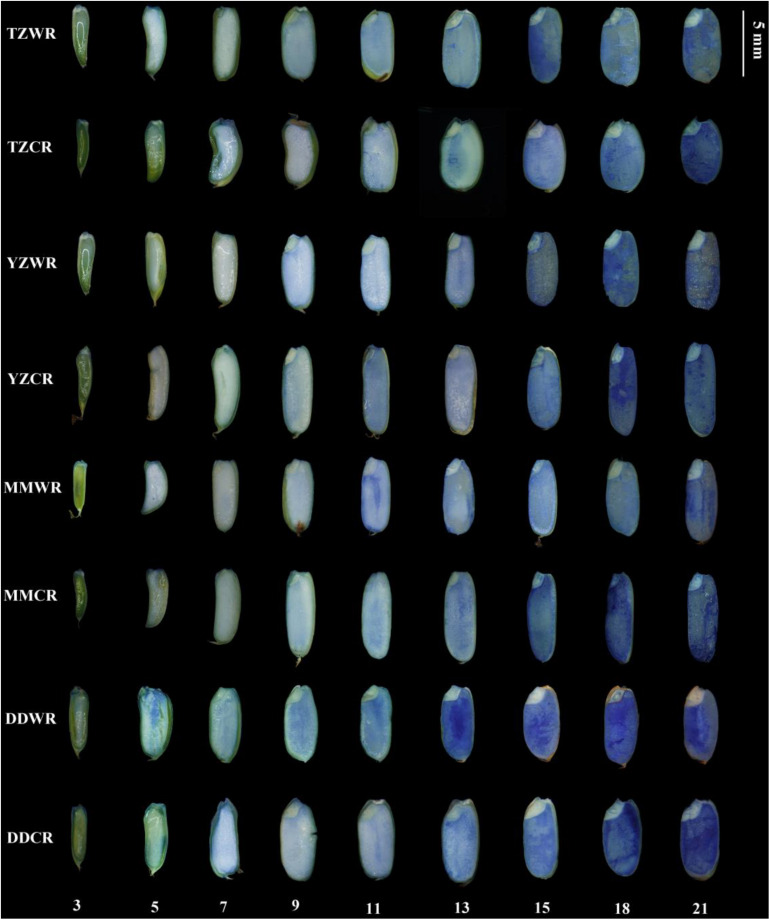
Evans blue staining in developing grains of weedy and cultivated rice. TZWR, weedy rice from Taizhou; TZCR, cultivated rice from Taizhou; YZWR, weedy rice from Yangzhou; YZCR, cultivated rice from Yangzhou; MMWR, weedy rice from Maoming; MMCR, cultivated rice from Maoming; DDWR, weedy rice from Dandong; DDCR, cultivated rice from Dandong. The Roman numerals at the bottom of the picture represent the days post anthesis.

Viability staining provides a means to follow the pattern and progression of cell death during endosperm development. Evans blue dye only stains dead cells, indicating a loss of membrane integrity and, consequently, viability. As endosperms of WR and ACR developed, their cells gradually dyed blue indicating a progressive loss of membrane permeability and cell death (Figure 3). The starch endosperm cells of TZWR were completely stained dark blue at 15 DPA, while those of TZCR were stained dark blue at 21 DPA. The starch endosperm cells of YZWR and MMWR were completely stained dark blue at 13 and 11 DPA, respectively, which was 2 days earlier than those of YZCR and MMCR. The endosperm cells of DDWR were completely stained dark blue at 11 DPA, which was 4 days earlier than those of DDCR (Figure 3). In all, compared to their ACR, the whole starch endosperm of WR was completely dyed dark blue by Evans blue 2–6 days earlier, implying that the endosperm cells of WR lost membrane permeability and died 2–6 days earlier (Figure 3).

#### TTC Dye Assay

**FIGURE 4:**
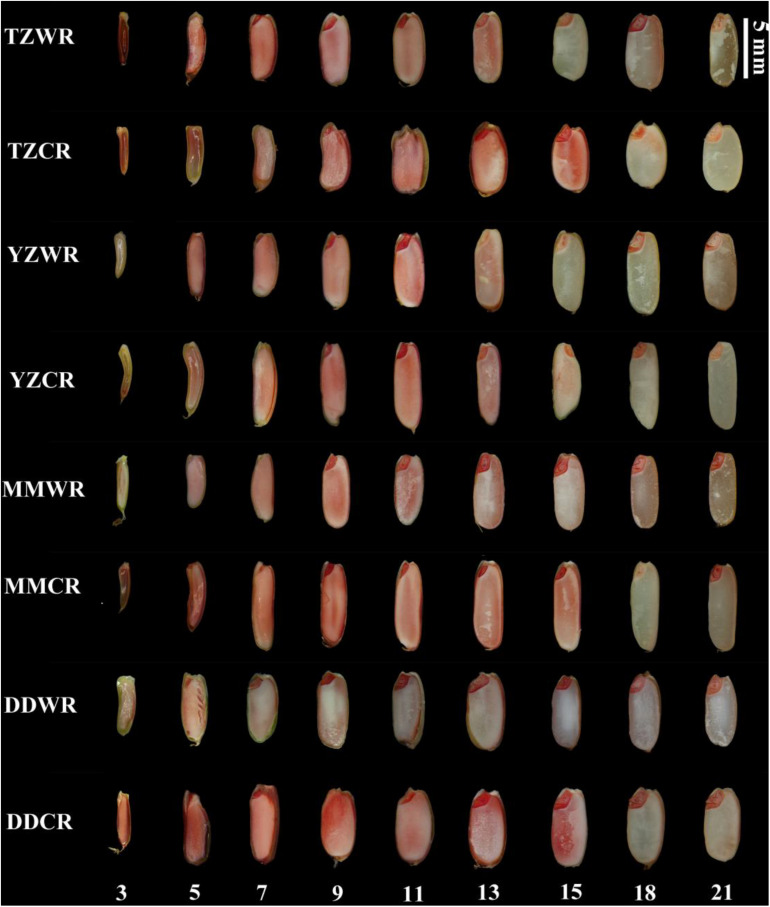
2,3,5-Tryphenyl tetrazolium chloride staining in developing grains of weedy and cultivated rice. TZWR, weedy rice from Taizhou; TZCR, cultivated rice from Taizhou; YZWR, weedy rice from Yangzhou; YZCR, cultivated rice from Yangzhou; MMWR, weedy rice from Maoming; MMCR, cultivated rice from Maoming; DDWR, weedy rice from Dandong; DDCR, cultivated rice from Dandong. The Roman numerals at the bottom of the picture represent the days post anthesis.

The endosperms of WR and their CR were decreasing to being dyed red by TTC with the development of the endosperm, which indicates that the endosperm cells gradually lost viability (Figure 4). The endosperm cells of DDWR were not dyed red at 9 DPA, while the endosperm cells of TZWR, YYWR, and MMWR could not be dyed red at 15 DPA. However, the endosperm cells of the ACR could not be dyed red at 18 DPA (Figure 4).

### Antioxidative Enzyme Systems of Endosperms of Weedy and Cultivated Rice

**FIGURE 5:**
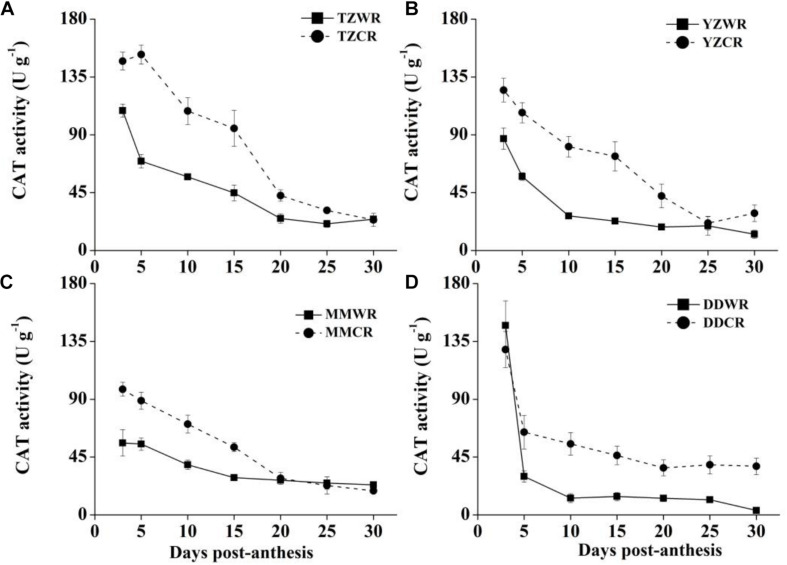
Changes in activities of CAT in weedy rice and cultivated rice. **(A)** Weedy and cultivated rice from Taizhou; **(B)** weedy and cultivated rice from Yangzhou; **(C)** weedy and cultivated rice from Maoming; **(D)** weedy and cultivated rice from Dandong. TZWR, weedy rice from Taizhou; TZCR, cultivated rice from Taizhou; YZWR, weedy rice from Yangzhou; YZCR, cultivated rice from Yangzhou; MMWR, weedy rice from Maoming; MMCR, cultivated rice from Maoming; DDWR, weedy rice from Dandong; DDCR, cultivated rice from Dandong. Solid line represents weedy rice and dash line represents cultivated rice. The data are presented as mean ± SE (*n* = 3).

**FIGURE 6:**
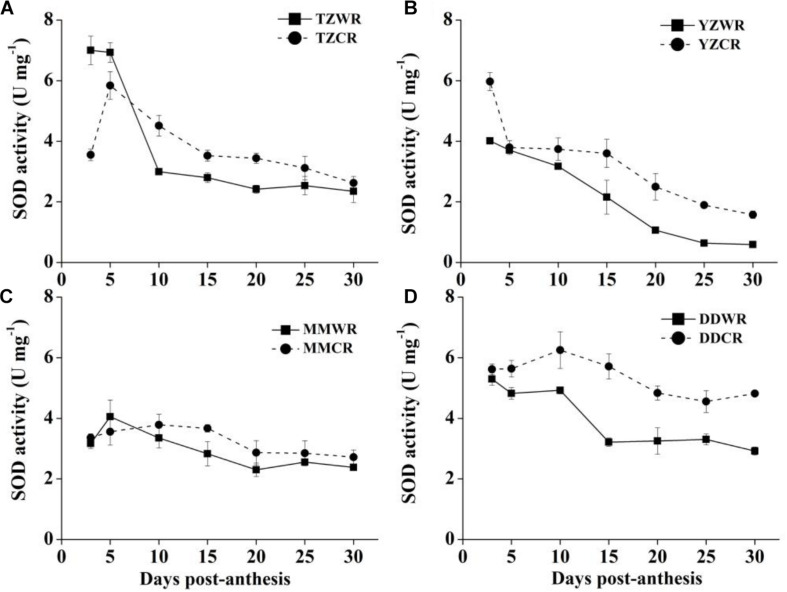
Changes in activities of SOD in weedy rice and cultivated rice. **(A)** Weedy and cultivated rice from Taizhou; **(B)** weedy and cultivated rice from Yangzhou; **(C)** weedy and cultivated rice from Maoming; **(D)** weedy and cultivated rice from Dandong. TZWR, weedy rice from Taizhou; TZCR, cultivated rice from Taizhou; YZWR, weedy rice from Yangzhou; YZCR, cultivated rice from Yangzhou; MMWR, weedy rice from Maoming; MMCR, cultivated rice from Maoming; DDWR, weedy rice from Dandong; DDCR, cultivated rice from Dandong. Solid line represents weedy rice and dash line represents cultivated rice. The data are presented as mean ± SE (*n* = 3).

**FIGURE 7:**
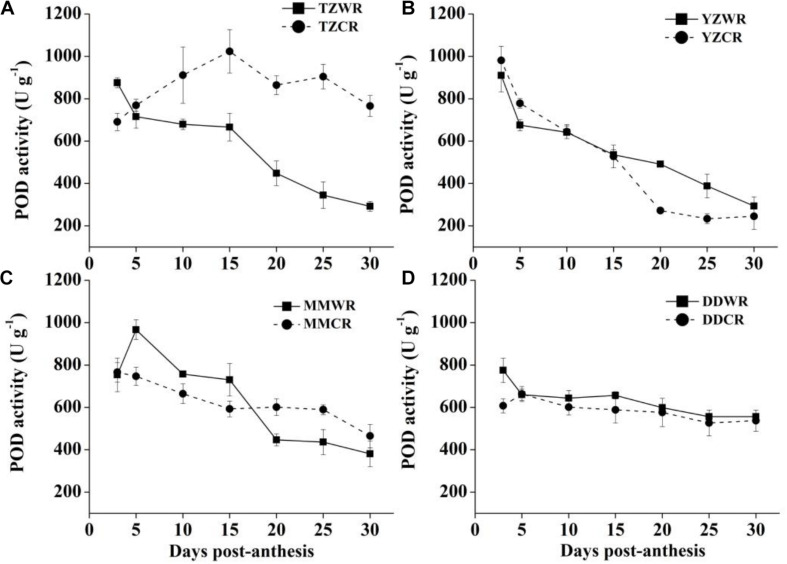
Changes in activities of POD in weedy rice and cultivated rice. **(A)** Weedy and cultivated rice from Taizhou; **(B)** weedy and cultivated rice from Yangzhou; **(C)** weedy and cultivated rice from Maoming; **(D)** weedy and cultivated rice from Dandong. TZWR, weedy rice from Taizhou; TZCR, cultivated rice from Taizhou; YZWR, weedy rice from Yangzhou; YZCR, cultivated rice from Yangzhou; MMWR, weedy rice from Maoming; MMCR, cultivated rice from Maoming; DDWR, weedy rice from Dandong; DDCR, cultivated rice from Dandong. Solid line represents weedy rice and dash line represents cultivated rice. The data are presented as mean ± SE (*n* = 3).

The antioxidative enzyme activity decreased gradually both in WR and their ACR, but in the earlier stage of endosperm development, the activity of WR was significantly lower than that of ACR (Figures 5–7).

#### CAT Activity

The CAT activity levels of TZWR were 10.39–82.95 U/g lower than that of TZCR at 3–25 DPA, while they were similar at 30 DPA between TZWR and TZCR (Figure 5A). The CAT activity of YZWR was 37.72–53.81 U/g lower than that of YZCR at 3–15 DPA, but there was no significant difference between those of YZWR and YZCR at 20–30 DPA (Figure 5B). The CAT activity of MMWR was significantly lower than that of MMCR at 3–15 DPA, but there was no significant difference between those of MMWR and MMCR at 20–30 DPA (Figure 5C). At 3 and 5 DPA, there was no significant difference between the CAT activity of DDWR and DDCR, and the CAT activity of DDWR was 23.62–42.20 U/g lower than that of DDCR at 10–30 DPA.

#### SOD Activity

The change trend of SOD activity of WR was similar to that of CR, and there was no significant difference between MMWR and MMCR (Figure 6C). The rate of decrease in SOD activity of WR from the other three geographical areas was faster than that of their respective ACR (Figure 6). The SOD activity of TZWR was the highest at 3 DPA, which was 3.45 U/mg higher than that of TZCR. The SOD activity of TZCR increased continuously after anthesis, reached a maximum at 5 DPA, and declined thereafter. The SOD activity of TZCR was 0.73–1.52 U/mg higher than that of TZWR at 10–20 DPA (Figure 6A). The SOD activity of YZWR and YZCR showed a downward trend, but the SOD activity of YZWR was 0.99–1.96 U/mg significantly lower than that of YZCR at 3, 25, and 30 DPA (Figure 6B). DDWR and DDCR had higher SOD activity at 3–10 DPA, and there was no significant difference between them. The SOD activity decreased at 15–30 DPA, but the decline rate of DDWR was faster, which made DDWR have significantly lower SOD activity than DDCR by 1.25–2.50 U/mg (Figure 6D).

#### POD Activity

The POD activity of TZWR showed a downward trend, peaking at 3 DPA, and was 184.53 U/g higher than that of TZCR. The POD activity of TZCR reached a maximum at 15 DPA and declined thereafter. The POD activity of TZCR was 357.52–559.19 U/g higher than that of TZWR at 15–30 DPA (Figure 7A). The POD activity of YZWR and YZCR showed a downward trend. The POD activity of YZWR was 103.25 U/g lower than that of YZCR at 5 DPA and was significantly higher than that of YZCR by 218.56 U/g at 20 DPA (Figure 7B). POD activity of MMWR was highest at 5 DPA, which was 219.77 U/g higher than that of MMCR, while the POD activity of MMCR peaked at 3 DPA; the POD activity of MMCR was 154.35 U/g higher than that of MMWR at 20 DPA (Figure 7C). There was no significant difference in POD activity between WR and ACR in Dandong (Figure 7D).

## Discussion

Cytological characteristics of endosperm cells involve the physiological process of cell death initiated and regulated by its own internal mechanism (Pennell and Lamb, 1997; Kabbage et al., 2017). During the rice endosperm development, the nucleus disintegrates first, but cells maintain high physiological activity after nuclear disintegration, and the grain weight continues to increase (Wu et al., 2016a, b). The mature endosperm of rice is composed of an aleurone layer and starch endosperm. The aleurone layer is an active tissue that stores proteins and lipids, while the starch endosperm is an inactive tissue that stores starch and protein (Zheng et al., 2017). Endosperm cells degrade and die, and the storage compounds accumulate constantly during the development of endosperm cells (Sabelli and Larkins, 2009; Wang et al., 2012; Kobayashi et al., 2013; Li et al., 2018). Evans blue staining shows that the death of endosperm cells occurs after anthesis until seed maturity (Young et al., 1997; Young and Gallie, 1999). In the current research, changes in cytological characteristics were found at early filling stage in WR and CR, and the cytological process was basically completed in endosperm tissues at the late grain filling stage (Figures 1–4). The endosperms of WR and CR still maintained dehydrogenase activity and cell activity after nuclear disintegration (Figures 1–4), which was consistent with previous studies. However, the nuclei of endosperm cells in WR were deformed and disintegrated earlier than those in ACR, and endosperm cells of WR lost activity earlier than those of ACR. This implied that the development of endosperm cells process in WR was faster than that in ACR, and this may be one of the important cytological mechanisms of rapid grain filling in WR.

The ROS burst, coming from the mitochondria, is result of the events leading to cell death and participating in the execution of cell death in plant cells (Breusegem and Dat, 2006). Antioxidative enzymes, such as SOD, POD, and CAT, can protect cells by scavenging ROS, and their activities are closely related to plant anti-aging (Corpas et al., 2006). Cheng et al. (2016) found that the accumulation of ROS is a direct cause of the acceleration of cell death in wheat endosperm and that the cytological progression can be alleviated by applying ROS scavengers. SOD, as the first enzyme involved in the scavenging of ROS, catalyzes the disproportionation of superoxide to produce H_2_O_2_, while CAT and POD transform H_2_O_2_ into water and oxygen (Corpas et al., 2006). The activities of SOD and CAT were higher in the grain during rice endosperm development (Zhang et al., 2015). In the current research, at least one antioxidant enzyme showed lower activity in WR than in its ACR from 5 to 15 DPA (Figures 5–7). Antioxidative enzyme activity is closely related to rice natural senescence and maturity. Short-cycle rice varieties had lower CAT and POD activities and senesced earlier than long-cycle varieties (Wang et al., 2010). Therefore, we speculate that the rapid cell development process in the endosperm of WR may be closely related to the activity of antioxidant enzymes. Under low antioxidant enzyme activity, cells cannot effectively scavenge oxygen free radicals, and cell macromolecules are poisoned, which accelerates the process of cell development in the endosperm of WR.

Compared with SOD and POD, CAT may play a more important role in scavenging ROS (Zhang et al., 2015). However, as the contents of ROS and malondialdehyde (MDA) in the endosperm of WR and ACR were not determined, the relationship between the cytological process and the ROS scavenging capacity of WR and ACR needs to be further verified. It has been reported that ascorbate peroxidase (APX), dehydroascorbic reductase (DHAR), glutathione peroxidase (GPX), glutathione reductase (GR), GSH, ASA, and other non-enzymatic substances can remove ROS, which may play an important role in regulating grain filling and the development process of endosperm cells (Yamauchi and Kusabe, 2001). The difference in the activity of these enzymes during endosperm development between WR and ACR will be the focus of future studies.

In our study, we found that the cytological process of grain in WR was faster and that the enzymatic activity for scavenging ROS was lower than that in ACR. Thus, it may be possible to extend the WR filling period by spraying chemical regulators that enhance antioxidant enzymes. Therefore, when the CR is mature and harvested, WR would be immature, decreasing its ability to disperse and facilitating its control. On the other hand, it was reported that ethylene can enhance the active oxygen system and stimulate free radical production in grains, and ethylene-induced H_2_O_2_ can reduce grain weight and the grain-filling period (Zhang et al., 2015; Chen et al., 2020). Chen et al. (2013) showed that spraying ethephon or 1-aminocyclopropane-1-carboxylic acid and methyl-glyoxal (bisguanythdrazone) inhibited grain filling. Based on our findings, we could speculate that WR may be induced to mature earlier and have a shorter filling period resulting in poor grain filling. Thus, WR would lose or decrease its germination ability.

The endosperm cells development process is closely related to grain filling, and the factors affecting grain filling can also affect the cytological process. There are many factors that affect the process of grain and endosperm cells development, such as sucrose unloading, transportation, and starch synthesis. The rapid transportation of sucrose from leaf and stem sheath to grain may be one of the reasons for the rapid development of rice grain (Hirose et al., 1997). Sucrose transporter (SUT) plays an important role in phloem loading and unloading. Therefore, we speculate that the expression of SUT related genes *OsSUT2*, *OsSUT3*, *Os-SUT4*, and *OsSUT5* in early stage of endosperm development of WR may be higher than that of CR. The sucrose synthetase and invertase regulate the decomposition and synthesis of sucrose, keep a certain sucrose concentration gradient between the library organ and phloem, which is conducive to sucrose transport into the seeds, and then synthesize starch in endosperm cells (Hirose et al., 2008). Therefore, the changes of sucrose synthase and invertase activities can directly affect the cytological process of endosperm cells (Yang and Zhang, 2006; Zhu et al., 2011; Zhang et al., 2015). Thirty-three major enzymes are reported to be involved in sucrose-to-starch conversion (SSC) during endosperm development in rice (Nakamura et al., 1989). Among these enzymes, sucrose synthase (SuSase, EC 2.4.1.13), acid invertase (AI, EC 3.2.1.26), ADP-glucose pyrophosphorylase (AGPase, EC 2.7.7.27), starch synthase (StSase, EC 2.4.1.21), and starch branching enzyme (SBE, EC 2.4.1.18) are considered to play key roles in this process (Nakamura et al., 1989; Wang et al., 2015). Many genes are involved in controlling the process of SSC, including *SuS2*, *SuS3*, *SuS4*, *OsCIN2*, *OsCIN4*, *OsINV2*, *AGPS1*, *AGPS2b*, *AGPL2*, *SSSIIa*, *SSSIIc*, *GBSSI*, *GBSSII*, and *SBEI* (Hirose et al., 2002; Ishimaru et al., 2005). We found that the cytological process of endosperm development in WR was faster than that in CR, and we speculate that the expression levels of sucrose synthesis related genes and starch synthesis related genes may be higher in WR at early endosperm development stage. The effect of sucrose transport and the expression of genes involved in SSC on endosperm cell development deserves further studied in the future.

Plant hormones play vital roles in regulating grain filling and the cytological process. Studies have shown that five kinds of hormones [auxin, gibberellin, cytokinin, abscisic acid (ABA), and ethylene] have significant effects on rice grain filling and endosperm development. Ethylene has been described to promote the onset of cell death in maize, whereas ABA works antagonistically (Young and Gallie, 2000a, b). It has been reported that the appropriate amount of ABA can improve the activities of starch synthesis related enzymes and gene expression, improve the grain filling rate (Zhu et al., 2011). In the process of rice grain development, higher ethylene content inhibited starch metabolism related enzyme activities (Yang and Zhang, 2006). In present study, we found that endosperm development and cytological process of WR were faster. We speculate that ethylene release rate, ABA content, and related gene expression of WR were significantly higher than those of CR at early grain development stage. The physiological mechanism of hormone regulating the rapid development of endosperm cytology in WR will be the focus of further research.

It is noteworthy that the programmed cell death (PCD) is also a genetically determined physiological process that plays an important role in the development of plant tissues, including endosperm (Pennell and Lamb, 1997; Fukuda, 2000; van Doorn et al., 2011; Fan et al., 2013; Domínguez and Cejudo, 2014; Xie et al., 2014; Locato and De Gara, 2018). PCD is a physiological process determined by PCD-related genes and plays an indispensable role in plant development (Schmid et al., 1999; Yin and Xue, 2012). In our study, we found that the nucleus degeneration rate and cytological process of endosperm cell nuclei in WR was faster than that in ACR, which may be related to PCD. Therefore, the differences in PCD-related genes, degradation of nuclear DNA, and other indicators related to the PCD process in endosperm cells between WR and CR need to be further studied.

## Conclusion

The endosperm cells of WR became degraded and lost viability earlier and more rapidly than those of their ACR. The ability of WR to scavenge ROS by endosperm cells was weaker than that of their ACR. The cytological process of endosperm cells in WR was faster than that in their ACR. The rapid cytological process shortened the grain filling period of WR and eventually led to the early maturity of WR. A better understanding of the mechanisms involved in the cytological process of endosperm cells will provide a theoretical and research-informed basis for understanding the weedy characteristics of WR.

## Data Availability Statement

The original contributions presented in the study are included in the article/Supplementary Material, further inquiries can be directed to the corresponding author/s.

## Author Contributions

XS and SQ conceived and designed the research. CZ and WX conducted the experiments and collected the data. CZ, WX, WD, and ZZ conducted field trials. CZ, HL, and XS analyzed the data. CZ and XS wrote the manuscript. All authors contributed to the article and approved the submitted version.

## Conflict of Interest

The authors declare that the research was conducted in the absence of any commercial or financial relationships that could be construed as a potential conflict of interest.

## Publisher’s Note

All claims expressed in this article are solely those of the authors and do not necessarily represent those of their affiliated organizations, or those of the publisher, the editors and the reviewers. Any product that may be evaluated in this article, or claim that may be made by its manufacturer, is not guaranteed or endorsed by the publisher.

## Abbreviations

WR: weed rice
ACR: associated cultivated rice
CR: cultivated rice
PCD: programmed cell death
TTC: 2,3,5-tryphenyl tetrazolium chloride
DAPI: 4′,6-diamidino-2-phenylindole
ROS: reactive oxygen species
CAT: catalase
POD: peroxidase
SOD: superoxide dismutase
DPA: days post anthesis
ACC: 1-aminocyclopropane-1-carboxylic acid.
