# Supplementary figures and images for: The Rapid Cytological Process of Grain Determines Early Maturity in Weedy Rice
Source: Front Plant Sci. 2021 Aug 31;12:711321. doi: 10.3389/fpls.2021.711321 (PMC8438156; doi:10.3389/fpls.2021.711321)

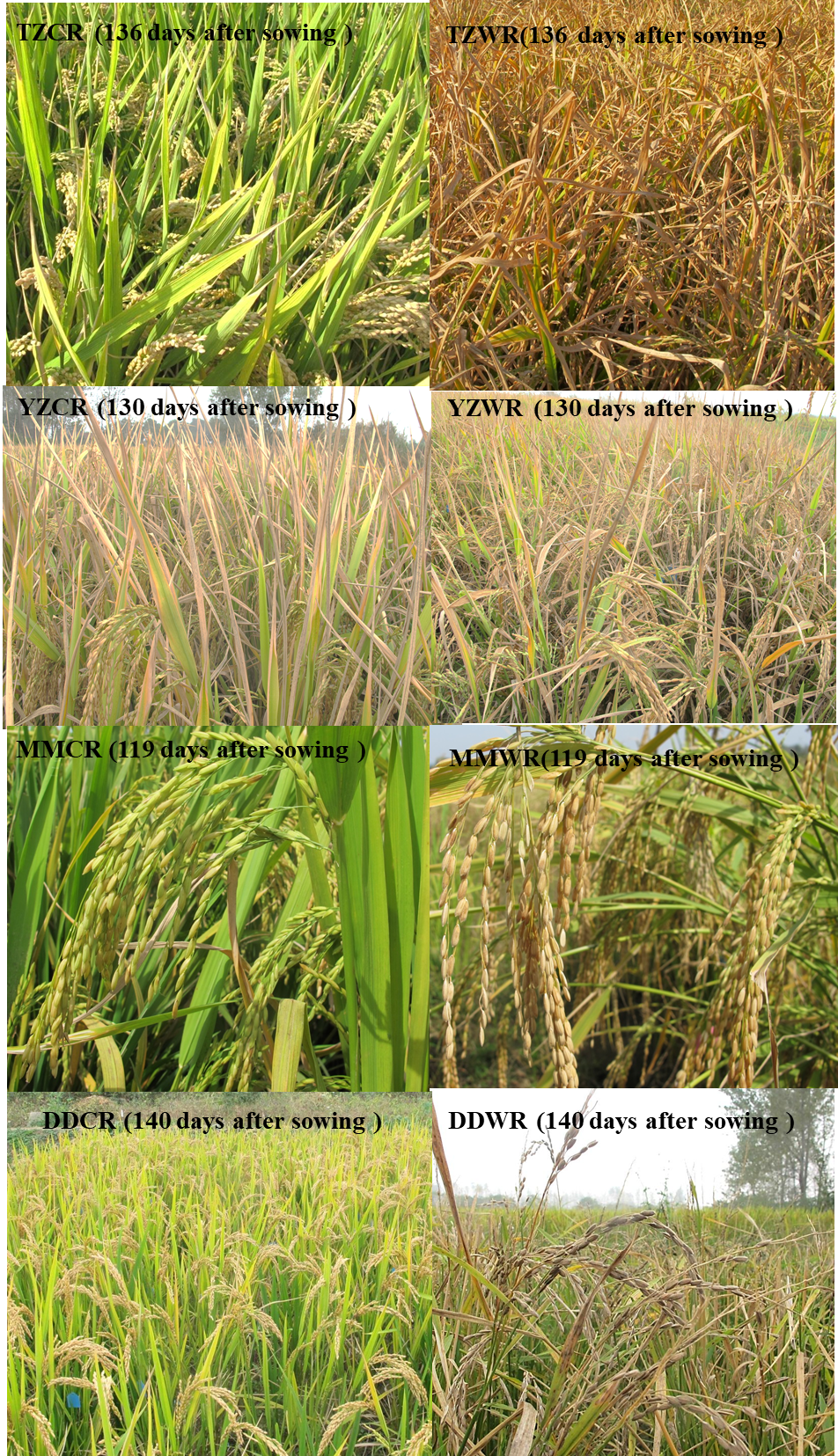


Supplementary Figure 1. The plant phenotype of weedy rice and cultivated rice.

Supplement: Supplementary file 1 [file Data_Sheet_1.docx]
